# The Pointing Errors in Optic Ataxia Reveal the Role of “Peripheral Magnification” of the PPC

**DOI:** 10.3389/fnint.2016.00027

**Published:** 2016-07-26

**Authors:** Philippe Vindras, Annabelle Blangero, Hisaaki Ota, Karen T. Reilly, Yves Rossetti, Laure Pisella

**Affiliations:** ^1^ImpAct Team, Lyon Neuroscience Research Center CRNL, INSERM U1028, CNRS UMR5292 and University Claude Bernard Lyon IVilleurbanne, France; ^2^Department of Experimental Psychology, University of OxfordOxford, UK; ^3^Department of Occupational Therapy, School of Health Sciences, Sapporo Medical UniversitySapporo, Japan

**Keywords:** optic ataxia, models, theoretical, mathematical modeling, posterior parietal cortex, visual central magnification, peripheral vision, visuo-motor control

## Abstract

Interaction with visual objects in the environment requires an accurate correspondence between visual space and its internal representation within the brain. Many clinical conditions involve some impairment in visuo-motor control and the errors created by the lesion of a specific brain region are neither random nor uninformative. Modern approaches to studying the neuropsychology of action require powerful data-driven analyses and error modeling in order to understand the function of the lesioned areas. In the present paper we carried out mixed-effect analyses of the pointing errors of seven optic ataxia patients and seven control subjects. We found that a small parameter set is sufficient to explain the pointing errors produced by unilateral optic ataxia patients. In particular, the extremely stereotypical errors made when pointing toward the contralesional visual field can be fitted by mathematical models similar to those used to model central magnification in cortical or sub-cortical structure(s). Our interpretation is that visual areas that contain this footprint of central magnification guide pointing movements when the posterior parietal cortex (PPC) is damaged and that the functional role of the PPC is to actively compensate for the under-representation of peripheral vision that accompanies central magnification. Optic ataxia misreaching reveals what would be hand movement accuracy and precision if the human motor system did not include elaborated corrective processes for reaching and grasping to non-foveated targets.

## Introduction

Optic ataxia is a rare and singular disease resulting from lesions of the posterior parietal cortex (PPC). Patients with optic ataxia have normal primary motor, proprioceptive, and visual functions but suffer from large visuo-motor dysfunctions (Perenin and Vighetto, 1988; Buxbaum and Coslett, 1998; Battaglia-Mayer and Caminiti, 2002; Jackson et al., 2009). Patients with optic ataxia make large spatial errors when pointing to peripheral targets in the contralesional visual hemifield (Perenin and Vighetto, 1988; Blangero et al., 2008). Previous studies have suggested that these “field effect” errors, observed only when the ipsilesional hand is used, are best expressed in oculo-centric reference coordinates (Khan et al., 2005a,b; Dijkerman et al., 2006). Further investigations have shown that unilateral optic ataxia patients exhibit systematic pointing errors toward the fixation point (Blangero et al., 2010) and that TMS over superior parieto-occipital cortex (SPOC) can induce similar reaching errors in healthy humans (Vesia et al., 2010). Optic ataxia patients make additional errors when pointing with their contralesional hand (hand effect). These errors have been attributed to impaired proprioceptive-motor transformations (Blangero et al., 2007) and are significantly reduced when visual feedback of the hand is provided, as in the procedure used by Blangero et al. (2010) whose data are modeled in the present paper.

While electrophysiology and brain imaging can demonstrate that a given structure contains information for ‘vision for action,’ brain lesions establish its crucial contribution to the processes of visuo-motor control. For example, it can be reasoned that if visuo-manual guidance is affected in optic ataxia, then its absence in these patients implies that it is crucial for ‘vision for action’ (Milner and Goodale, 1995; Pisella et al., 2000; Gréa et al., 2002). However, patients with optic ataxia following damage to the PPC are still able to guide actions toward visual objects in everyday life (Rossetti et al., 2003; Gaveau et al., 2008). Moreover, with this basic neuropsychological approach, almost the whole brain may appear crucial for ‘vision for action,’ since some aspects of visuo-motor control are impaired in many clinical conditions, for example hemineglect (Heilman et al., 1983; Mattingley et al., 1998), cerebellar ataxia (Day et al., 1998; Frassinetti et al., 2007), Parkinsons disease (Viviani et al., 2009), and even in patients with lesions of the ventral visual processing stream (Hesse et al., 2012).

Jeannerod (1988) proposed that each neural substrate implements a different processing step in a chain of visual coordinate transformations followed by visuo-motor transformations. Another view suggests that visually guided actions rely on multiple independent cortical and sub-cortical visual-to-motor pathways (Rossetti et al., 2000; Pisella et al., 2006; Gaveau et al., 2014). While the in-built redundancy of multiple independent visuo-motor pathways provides the system with protection against a lesion, in fact, each system cannot perfectly substitute for the others. Even though only lesions of primary motor cortex and its descending tracts fully prevent reach and grasp to visual objects, other lesions in the visual-to-motor network lead to characteristic errors. This suggests that different and complementary neural structures may have evolved to permit efficient and accurate visuo-motor control in a larger panel of behavioral contexts (Rossetti and Pisella, 2002; Heed et al., 2011; Granek et al., 2013). The neuropsychological challenge then becomes to define when [in which spatial, temporal, and cognitive condition(s)] their specific contribution to visuo-motor control is crucial.

Thanks to more than 20 years of detailed observation of the exact conditions in which patients with optic ataxia are impaired such spatial, temporal, and cognitive characterisation is now available for the PPC. The PPC is involved when peripheral vision of the hand or the target has to be integrated on-line for goal-directed actions (Rossetti et al., 2003; Pisella et al., 2006, 2009; Granek et al., 2012). Interaction with visual objects in the environment requires an accurate correspondence between visual space and its internal representation within the brain. Ocular fixation allows the object’s position in space to be matched with the center of each retina (the fovea) and thereby provides accurate object position information through the proprioception of the ocular muscles (to be integrated with respect to the head and trunk positions, with the possibility that the hand position can also be coded in this body reference frame). However, spontaneous eye-hand coordination reveals that hand movements and eye movements are actually planned in parallel based on peripheral visual information (Prablanc et al., 1979; Biguer et al., 1982; Gaveau et al., 2008), but visuo-motor planning based on peripheral visual information is inaccurate (Prablanc et al., 1979). The larger receptive fields in the peripheral retina are reflected by gross visuo-spatial representation in peripheral vision. Moreover, the center of the retina (the fovea) contains a high density of photoreceptors with small receptive fields and provides high visual acuity. Outside the fovea, however, this density drops abruptly, and with it, acuity in peripheral vision. This inhomogeneous receptor density on the retina is reflected by magnification of central vision (and under-representation of peripheral vision) in the visual spatial representations of the superior colliculus and the occipital cortex (Schwartz, 1980; Ottes et al., 1986; Van Gisbergen et al., 1987; Polimeni et al., 2006; Schira et al., 2007; Wu et al., 2012).

During evolution, some neural structures have probably developed to implement specific processes for ‘correcting’ or ‘refining’ the basic motor output prepared by more ancient brain structures, in particular for correcting peripheral under-representation. Despite the presence of basic visual errors due to the inaccuracy of spatial information from peripheral vision, hand movements end accurately on the visual target thanks to visuo-motor control processes which automatically ‘correct’ the initial movement parameters (Goodale et al., 1986; Pélisson et al., 1986; Pisella et al., 2000) before (if movement onset time is not constrained), or during movement execution. These fast visuo-motor control processes are impaired in optic ataxia patients (Milner et al., 1999; Pisella et al., 2000; Gréa et al., 2002; Rossetti et al., 2005; Blangero et al., 2008) and are therefore thought to rely on the PPC. The PPC could, therefore, be the recently evolved neural system that has a specific role in compensating for the inaccuracy of spatial information from peripheral vision. Notably, the PPC contains an area V6a, within the occipito-parietal sulcus, which in monkeys and humans has the most accurate spatial representation of the visual world, with an over-representation of the periphery compared with other visual areas (Galletti et al., 1996, 1999; Pitzalis et al., 2013). The dorsal stream of visual processing (medio-dorsal occipito-parietal pathway) may thus be actively involved in building the sole accurate interface between vision and action able to compensate for the spatial under-representation of peripheral vision that emerges from the structural organization of the retina.

The pointing errors of optic ataxia patients increase with target eccentricity and are directed toward eye fixation (Blangero et al., 2010), as if target location in peripheral vision is increasingly under-estimated with increasing target eccentricity. We therefore make the hypothesis that the mechanisms that allow movements toward visual objects in the periphery to be accurate are those that compensate for the spatial under-representation of peripheral vision. If this is true, the pointing errors made by optic ataxia patients with the ipsilesional hand toward the contralesional visual field (field effect errors) should be stereotypical and reflect the spatial distortion (gradual under-representation of the periphery) characteristic of the vicarious structure(s) guiding the pointing movement in the absence of the PPC.

## Materials and Methods

### Subjects

The performance of seven optic ataxia patients (five men and two women aged from 38 to 75 years old, mean age 58 years old, four with left hemisphere lesions and with three right hemisphere lesions; demographic and clinical information available in Table 1 of Blangero et al., 2010) was compared to that of seven control subjects (five men and two women) aged from 32 to 76 years old (mean age 52 years old) who had with no history of neurological disorders. The superimposition of the lesions of our seven patients delimited a site located in the posterior parietal deep white matter in-between the intraparietal sulcus and the parieto-occipital sulcus; the lesions spared the precuneus (Blangero et al., 2010).

**Table 1:** Results of control subject analyses.

	Mixed-effects mode (8 param.)				Individual models (5 param.)					
		
Significant parameters	Value	Confid. Interv	LLR	Prob.	Average	Minimum	Maximum	%SS red.	K-S	Prob
Contraction error CE (fixed)	-0.032	[-0.041, -0.019]	15.79	7.1^∗^10^-5^	-0.032	-0.047	-0.012	22.3	0.949	9.1^∗^10^-10^
Contraction error CE (SD)	0.010	[0.005, 0.019]	22.39	2.2^∗^10^-6^	0.013	-	-	-	-	-
Hemifield bias HFB (fixed)	0.802	[0.658, 0.946]	108.54	<10^-12^	0.822	0.47	1.25	16.5	0.923	1.7^∗^10^-8^
Vertical bias Y_0_ (SD)	0.376	[0.209, 0.675]	40.41	2.1^∗^10^-10^	-0.11	-0.62	0.39	8.7	0.699	3.0^∗^10^-4^
Horiz. bias X_0_ (right hand)	0.224	[0099, 0.507]	16.12	5.9^∗^10^-5^	0.23	-0.21	0.43	5.9	0.768	3.8^∗^10^-5^
Horiz. bias X_0_ (left hand)	0.453	[0.235, 0.874]	18.9	1.4^∗^10^-5^	-0.29	-0.97	0.31	5.0	0.468	3.2^∗^10^-2^
Expon. variance slope	0.012	[0.006, 0.018]	14.74	1.0^∗^10^-4^				-	-	-


*The four first columns indicate the value, confidence interval, Log-Likelihood Ratio (LLR), and probability of the parameters of the best fitting model for controls’ errors. The six last columns indicate the results of individual analyses and their assessment by Kolmogorov-Smirnov (KS) tests (see Materials and Methods). There is no individual result for the standard deviation (SD) of contraction errors (CE) because individual models fit a single CE by individual. However, an estimate of the SD of CE (0.010) can be computed as the standard error of the fitted individual values (0.013). For the same reason, individual models cannot assess the two parameters describing the residual errors in mixed effect models, i.e., the variation of residual errors with target eccentricity and the estimate of the residual variance. The later parameter is not included in the table because it is never tested in ME analyses. The significance of the exponential variance slope (last row in the table) indicates that residual variance increases with target eccentricity in an exponential manner and describes the variation of residual errors better than other standard functions such as powers of target eccentricity.*

The seven control subjects gave informed oral consent to participate in the experiment according to the French law (4 March 2002) on human subjects’ rights. This group study was based on non-invasive routine tests performed for the clinical diagnosis of optic ataxia (see Perenin and Vighetto, 1988) in two university hospitals (Tohoku University Hospital, Sendai, Japan, and Hôpital Henry Gabrielle, Hospices Civils de Lyon, Lyon, France) and one private hospital (Nakamura Memorial Hospital, Sapporo, Japan).

### Experimental Design and Procedure (See Blangero et al., 2010)

A vertical Perspex screen (1 m high, 1.5 m wide) was placed 30 cm in front of the subjects. On the side facing the subject there was a permanent fixation point marked at the center of the screen. Using a chair with adjustable height the subject was always placed with his/her body midline aligned with, and his/her eyes at the same height as, the central fixation cross. On the other side of the screen, facing the experimenter but invisible to the subject, was a translucent A0 sheet of tracing paper that displayed a matrix to guide the manual laser presentation of the visual targets and to record the endpoints of each reaching movement. The targets (3 mm diameter red laser light points) were presented on the left or right side (relative to the subject) of the fixation point at ±50, 110, 170, and 250 mm in X-coordinates, which corresponded to ±10°, 20°, 30°, or 40° of visual angle along the horizontal meridian (*Y* = 0 mm). In addition to being presented at fixation level (*Y* = 0 mm), stimuli were also presented at the same X-coordinates but 20° above and below (*Y* = ±110 mm). Thus, a total of 3 × 4 target points were presented in each visual field. The order of presentation of visual targets on this (X,Y) matrix was randomized with respect to visual field and location, whereas the use of the left or right hand was tested in blocked conditions.

Subjects were instructed to maintain fixation at the center fixation location and point with their index finger to a target as soon as they saw it. They were asked not to correct their responses after pointing. The pointing movements were performed in the light, thus vision of the hand was provided throughout the trial, but no visual feedback of their final error was provided. The experimenter first positioned the laser pointer on a given target position, he then checked the subject’s eye fixation, and finally switched the laser on. Then he switched the laser off (after ∼1 s) and continued to check ocular fixation while the subject was moving his/her hand. The subject was instructed to keep the finger in contact with the reach panel (without correcting the position) until the experimenter instructed her/him to move it back. Landing points for each pointing movement were marked by drawing a line between the landing point and the target. These lines were invisible to the subject.

### Data Collection

Before the testing, we prepared a large A0 sheet of tracing paper on which all target positions, the central fixation cross and the X and Y axes were carefully marked for the experimenter and so that they would remain unseen by the subject. We used two sources of light symmetrically positioned with respect to the subject. The experimenter marked the center of the intersection of the two finger shadows on the tracing paper. After each experiment, the translucent tracing paper was positioned over a millimetric graph paper, matching carefully the fixation cross and the target matrices of the two sheets. The exact X, Y coordinates were thus read off-line.

### Pointing Data Analysis

The quantitative characterization of both patient and control subject pointing errors was carried out with non-linear mixed-effect (ME) analyses (nlme package with R version 2.14.1). ME analyses are based on multilevel (hierarchical) modeling (Snijders and Bosker, 1999; Pinheiro and Bates, 2006; Hox, 2010). As such, they are the best statistical method to reveal significant differences between groups (controls vs. patients), between individuals within groups, and between hands or hemifields within individuals. These differences may concern both fixed and random effects. Fixed effects indicate significant differences between group averages. Random effects indicate significant differences between within-group inter-individual variability. Therefore, ME analyses can reveal between-group differences for both the average value and the inter-individual variability of model parameters. Another advantage of ME analyses is that they allow residual errors to be modeled as a function of model parameters. This is especially interesting because the variability in the residual errors may increase with target distance and may depend on groups or hemifields. In general, residual error models involved 2 or 3 parameters. As a result, ME models had 2 or 3 more parameters than the corresponding individual models.

Mixed effects analyses have two main drawbacks. First, they may yield excessive type I error rates when the sample size is smaller than 30 (Maas and Hox, 2005; Paccagnella, 2011; Vindras et al., 2012). As it was impossible to increase the sample size due to the scarcity of patients with Optic Ataxia, we used a low significance threshold (10^-4^ instead of the usual 0.05), and we checked the results of the ME analyses by carrying out individual linear analyses and testing their outcome using three complementary methods. First, to confirm whether a fixed or random effect was significant, we tested the samples of *p*-values provided by matching individual linear models using a new method based on the Kolmogorov–Smirnov test (Vindras et al., 2012). This test yields a significant outcome not only if the inter-individual average is significantly different from zero (ME Fixed effect), but also if an abnormally large number of individual analyses indicate significant negative or positive effects (ME Random Effect). Second, we confirmed significant between-group differences revealed by ME analyses by using Mann–Whitney *U* tests to assess whether individual parameter values differed between groups. Third, when ME analyses revealed between-hand or between-hemifield differences, we used non-parametric Wilcoxon signed-rank tests to assess the hand-specific or hemifield-specific parameters provided by the individual models.

The second drawback of ME analyses is their complexity, and because of this, stepwise forward modeling is strongly recommended (Pinheiro and Bates, 2006). Thus, throughout these data-driven analyses we introduced parameters one by one into the models, keeping the most significant ones at each step. This process means that significant parameters cannot be described in advance but are presented throughout the results section. Because our goal was to check for differences between controls and patients and hand- and hemifield- specific differences within patients, the data set was progressively enriched in four stages. We first modeled the errors made by control subjects. Second, we fit the errors made by patients pointing in the ipsilesional hemifield with their ipsilesional hand by introducing new parameters in a stepwise manner to test whether and how these errors differed from control subjects’ errors. Third, we fit the errors made by patients pointing in the ipsilesional hemifield with their contralesional hand to test whether and how these errors differed from the previous data set (including errors made both by control subjects and patients with their ipsilesional hand in their ipsilesional hemifield). Finally, we modeled the additional errors specifically associated with the targets in the contralesional hemifield which consisted exclusively of large errors directed toward the point of ocular fixation (Blangero et al., 2010) that depended non-linearly on target eccentricity. These were modeled using one or two-parameter equations. These equations were standard modeling functions (polynomial, logarithmic, or power functions), as well as complex logarithms based upon those used in the literature to describe the collicular and cortical visual mappings (Schwartz, 1980; Ottes et al., 1986; Van Gisbergen et al., 1987; Polimeni et al., 2006; Schira et al., 2007; Wu et al., 2012). Section “Results” progressively describes the outcome of these four stages.

## Results

**FIGURE 1:**
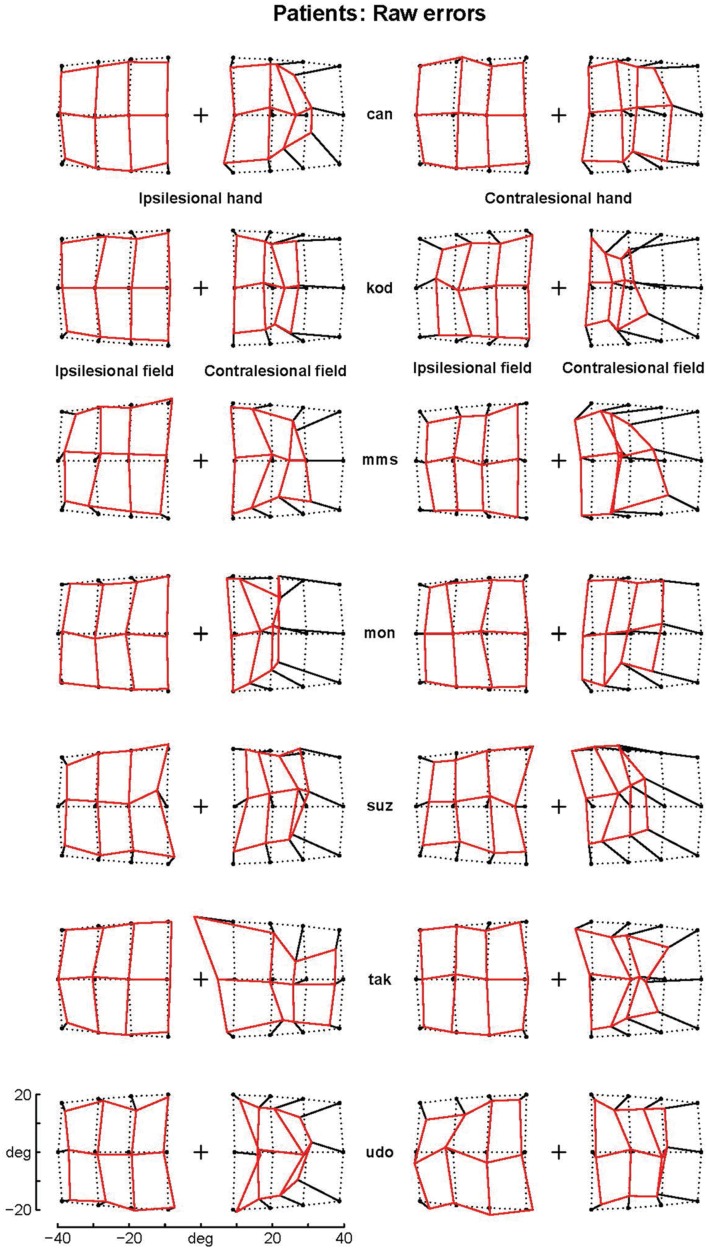
**Patients’ raw errors.** The pointing errors for the ipsilesional (left columns) and contralesional hand (right columns) are shown by segments starting joining the targets (black point) to the endpoint. Endpoints are joined by segments into a continuous grid to enhance the deformation with respect to the targets grid (dotted points). Note that the errors in the contralesional hemifield (second and forth column) are much larger than those in the ipsilesional hemifield and display a stereotypical centripetal pattern. For sake of clarity, the data from patients with right lesions (suz, tak and udo) have been inverted by symmetry with respect to a central vertical axis.

**FIGURE 2:**
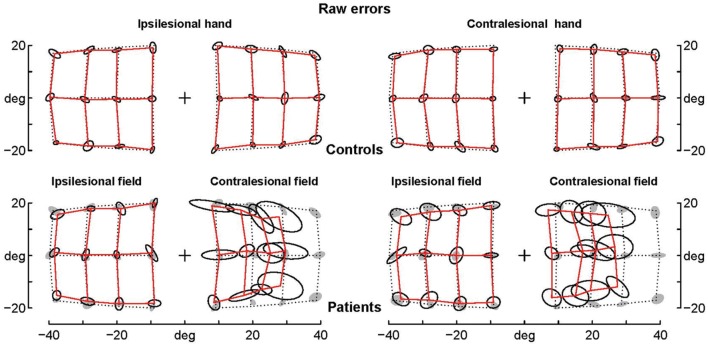
**Average raw errors.** Average raw errors are shown for controls **(top)** and patients **(bottom)** together with the ellipses of inter-individual variability. Controls’ ellipses are also shown as shadows in the patients’ error grid. Note that for the contralesional hemifield patients’ average endpoints are far away the controls’ ellipses, while for the ipsilesional hemifield patients’ average endpoints are often within the controls’ ellipses, which suggests that they are not significantly different.

**FIGURE 3:**
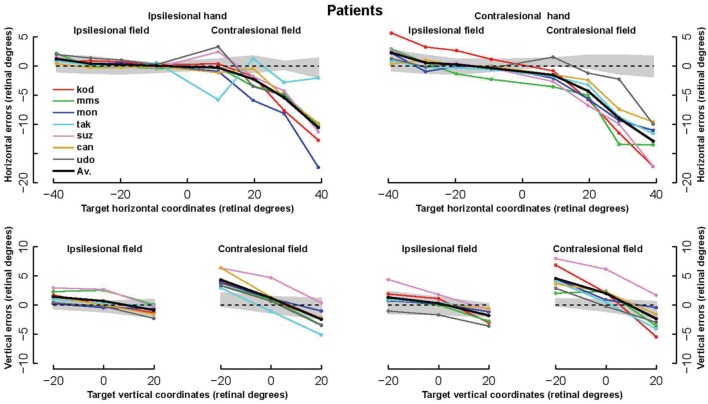
**Horizontal and vertical errors of patients.** **(Upper row)**: The horizontal component of errors is represented as a function of the target horizontal coordinate from the left of the ipsilesional hemifield to the right of the contralesional hemifield. **(Bottom row)**: Vertical component of errors represented as a function of target vertical coordinate. For each hand, left and right panels display errors in the ipsi- and contra-lesional hemifield, respectively. Note the highly stereotypic pattern of the horizontal error components, especially with the ipsilesional hand (left) where the average across all subjects (bold line) hides almost completely four individual lines.

The patients’ final pointing positions are displayed in **Figure 1.** While there are some individual differences, overall their behavior is strikingly similar. In the ipsilesional visual hemifield (columns 1 and 3) patients were often as accurate as controls, although group averages (**Figure 2**) reveal that patients had higher inter-individual variability and tended to point closer to the fixation point (central cross) than controls. In the contralesional hemifield (**Figure 1**, columns 2 and 4), nearly all patients displayed the same pattern of large errors with both hands. The pattern is characterized by errors directed toward the fixation point which increase non-linearly with target eccentricity (**Figure 2**). The patients’ stereotypic pointing behavior is best visualized by representing errors in the horizontal axis as a function of the target’s horizontal coordinate (**Figure 3**, top). This stereotypical pattern strongly suggests that patients with unilateral superior parietal lesions use the same vicarious or degraded visuomotor process to point to targets in the contralesional visual hemifield, regardless of the pointing hand. The goal of this study was to characterize this visuomotor process using quantitative models in order to get an insight into the contribution of the PPC to the planning and execution of movements toward visual targets.

To achieve this goal we carried out a three-stage analysis. First, we modeled the errors made by control subjects. Second, we enriched this model with terms characterizing the differences between the patients and controls in the ipsilesional hemifield. Third, we further enriched the model with terms fitting the additional errors made by patients in the contralesional hemifield. For each stage, we use mixed-effect (ME) multilevel (hierarchical) analyses in order to reveal significant differences between groups (control vs. patients), between individuals within groups, and between hands or hemifields within individuals. As ME models may yield excessive type I error rates when the sample size is smaller than 30, we used a low significance threshold (10^-4^ instead of 0.05) and checked the results by linear individual analyses.

### Control Subjects

Control subjects made highly significant pointing errors that were best fitted by a 5-parameter model (Equation 1). In this model, only the hemifield bias (HFB) parameter remained constant across subjects, hands, and hemifields (fixed component with no significant random component in ME analyses). The stability of this effect suggested to us that it could be a systematic bias due to the measurement procedure or a small systematic error. Although it was statistically significant (see **Table 1**), the magnitude of this horizontal bias was equal to only +0.8° in the right hemifield and -0.8° in the left hemifield. The second most significant parameter was the contraction error (CE). This CE did not vary across hand or hemifield, and was purely linear (no quadratic component). Its inter-individual average was equal to -0.032. It displayed small but significant variations across individuals (random component, standard error 0.010, see **Table 1**; values from -0.012 to -0.047 according to individual analyses). The three other parameters were not significantly different from 0 at the group level, but displayed significant individual variations. They included a vertical bias Y0 that was constant across hands, hemifields, and targets, as well as a horizontal bias X0 with significant hand-dependent differences that varied across subjects (HDB bias). The models defined by Equations 1 and 2 fitted the data as well as each other with the same number of parameters. In Equation 2, the contraction bias applies to the vector from the fixation point (0,0) to the target whereas in Equation 1 it applies to the vector from the point (-X0 ± HDB, Y0) to the target. Although Equation 2 was simpler, we took Equation 1 for the baseline model because it provided slightly better results than Equation 2 when the patient data were progressively introduced.

$$[\begin{array}{c} errx\\ erry \end{array}]=\left[\begin{array}{c} X_{0}\pm HFB\pm HDB\\ Y_{0} \end{array}\right]+CE\times \left[\begin{array}{c} tgtx+X_{0}\pm HDB\\ tgty+Y_{0} \end{array}\right]$$

$$[\begin{array}{c} errx\\ erry \end{array}]=\left[\begin{array}{c} X_{0}\pm HFB\pm HDB\\ Y_{0} \end{array}\right]+CE\times \left[\begin{array}{c} tgtx\\ tgty \end{array}\right]$$

The mixed-effects model based on Equation 1 explained 34% of the sum of square of 336 2D-errors with eight parameters (X0, Y0, CE, HDB, HFB, SD(CE) and two parameters describing the residual errors). The matching 5-parameter individual models explained 21 to 51% (36 % on average) of the 96-degrees-of-freedom sum of square errors (48 2D-errors by individual). These individual analyses confirmed the results of the hierarchical mixed effect model. Indeed, as shown in **Table 1**, all significant parameters uncovered by the mixed effect analyses were also significant according to the Kolmogorov–Smirnov test on the results of the individual analyses.

### Patients’ Pointings to Targets in the Non-ataxic (Ipsilesional) Hemifield

When pointing to targets in their non-ataxic field with both their ipsi- and contra-lesional hand, the patients made the same kinds of errors as controls. They displayed highly significant CEs, horizontal biases, and vertical biases that were all confirmed by Kolmogorov tests of individual parameters (CE: *k* = 0.9987, *p* = 0; vertical bias: *k* = 0.4454, *p* = 0.0439; horizontal bias: *k* = 0.7109, *p* = 0.0002) with the exception of the between-hand difference for the horizontal bias (*k* = 0.324, *p* = 0.187).

Analyses comparing model parameter values for controls and patients revealed two differences (**Table 2** for ME analyses and **Table 3** for individual analyses). First, patients pointing to targets in their ipsilesional hemifield made larger CEs than controls with both their ipsilesional and contralesional hands. In addition, when pointing with their contralesional hand, patients showed higher inter-individual variability in CEs than controls. Second, for both hands, the residual errors were larger for patients than for controls. Moreover, the residual errors increased more with target eccentricity for patients with the contralesional hand than for controls and patients with the ipsilesional hand. Finally, comparisons between the ipsi- and contra-lesional hands of patients revealed that the CEs and the residual errors were larger with the contralesional hand than with the ipsilesional hand (Wilcoxon tests, see **Table 3**). In summary, in their ipsilesional field patients pointed with errors that were similar to controls, although they had larger contraction and residual errors. As a result, only three additional parameters in the control model were needed to fit 118 additional 2D-errors.

**Table 2:** Mixed-effects analyses of patients’ pointings in the non-ataxic field.

	Ipsilesional hand			Contralesional hand			Both hands		
			
Parameters	Value	LLR	Prob.	Value	LLR	Prob.	Value	LLR	Prob.
Contraction error (fix ed)	-0.053	**7.73**	**0.0054**	-0.069	**12.78**	**3.5^∗^10^-4^**	-0.063	**12.79**	**0.0015**
Contraction error (SD)	0.170	0.18	0.6692	0.046	**13.06**	**3.0^∗^10^-4^**	0.031	2.74	0.0977
Hemifield bias (fix ed)	0.570	2.37	0.1237	1.270	1.98	0.1591	0.730	0.35	0.5548
Horiz. bias (SD interindiv)	na	na	1	0.760	1.1	0.2935	0.000	0.00	0.9988
Vertical bias (SD)	0.976	5.314	0.0212	0.977	6.56	0.0105	0.874	4.19	0.0406
Variance ratio	1.230	**11.4**	**0.0007**	1.520	**48.35**	**3.6^∗^10^-12^**	1.430	**55.78**	**8.1^∗^10^-14^**
Ex pon. variance slope	0.012	6.05	0.0139	0.021	**49.72**	**1.8^∗^10^-12^**	0.017	**46.61**	**8.7^∗^10^-12^**
Ex pon. variance + ratio	-0.0012	4.51	0.0336	0.016	**7.98**	**4.7^∗^10^-3^**	0.007	0.73	0.3905


*In the first three columns, the data set included all control subject pointings as well as patients’ pointings with the ipsilesional hand in the ipsilesional field. The significance tests consisted of comparing one model with separate parameters for the patients and controls to a simpler model with the same parameters for both groups. In the last six columns, the data set included all control subject pointings as well as patients’ pointings with the both the ipsilesional and contralesional hands in the ipsilesional field. In columns 4–6, the patients’ pointings with the contralesional hand had a different parameter that the pointings including controls and patients’ pointings with the ipsilesional hand. In columns 7–9, the patients’ pointings had a different parameter from all control subject pointings. The three last rows of the table characterize residuals errors. They can be larger in a specific group (variance ratio), or increase more with target eccentricity in a specific group (Exponential variance slope, seventh line), or both (Exponential Variance slope and variance ratio). All these comparisons were tested separately for each parameter.*

*Bold characters indicate significant values at the 0.01 threshold.*

**Table 3:** Individual analyses of patients’ pointings in the non-ataxic field.

	Ipsilesional hand			Contralesional hand			Both hands			Unilat. paired comp.		
				
Significant parameters	Value	*U*	Prob.	Value	*U*	Prob.	Value	*U*	Prob.	Value	W	Prob.
Contraction error (average)	-0.053	41	0.038	-0.083	42	0.0262	-0.068	43	0.0175	0.030	25	00391
Horiz. Bias (left hand average)	-0.786	31	0.456	-	-	-	-0.067	34	0.2593	0.526	24	0.5470
Horiz. Bias (right hand average.	-	-	-	-1.312	36	0.1649	-0.394	34	0.2593	-	-	-
Vertical bias (average)	0.401	13	0.318	-0.075	18	0.4557	-0.192	14	0.2086	0.476	19	02344
Variance ratio	1.598	15	0.259	2.325	1	0.0012	2.117	3	0.0041	1.477	3	0.0391


*Parameters fitted by linear modeling of individual data are compared using either Mann–Whitney *U* test (control-patients comparisons) or Wilcoxon’s signed-rank test (patients’ ipsilesional vs. contralesional hand). These complementary analyses confirm the differences of contraction error and residual variance evidenced by mixed-effects analyses (**Table 2**).*

### Patients’ Pointings to Targets in the Ataxic (Contralesional) Hemifield

**FIGURE 4:**
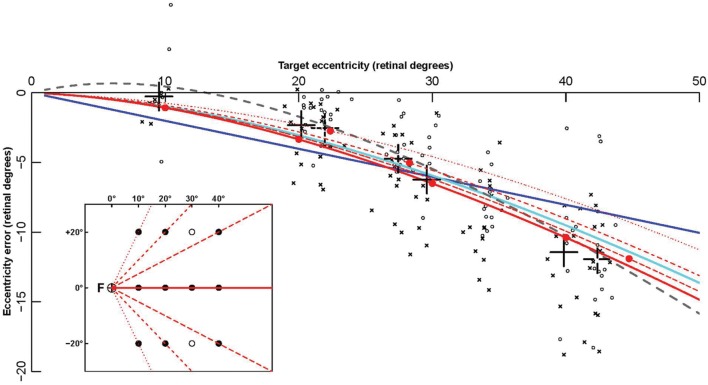
**Fitting of contralesional hemifield data points by eccentricity functions.** The eccentricity errors (defined as final endpoint position minus target retinal eccentricity) are shown as a function of target retinal eccentricity. Data points are computed from patients’ pointing in the contralesional hemifield made with the ipsi- (circles) and contralesional (crosses) hand. The large black crosses represent the average of these endpoints for the different targets (for each X coordinate, endpoints toward targets at *Y* = -20 and *Y* = +20° were grouped together). Colored lines show some of the fitting functions in **Table 4**: the linear function F_01_ (Blue), the logarithmic function F_06_ (cyan), the power function F_14_ (gray), and the complex logarithmic function F_08_ (red). Solid red dots represent the eccentricity errors predicted by F_08_. In contrast with all other functions, that depend on retinal eccentricity only, F_08_ results are also modulated by target retinal direction. The function is shown for the 0° horizontal direction (red continuous line), and for three other directions (±63°: dotted line, ± 45°: dashed line, ± 27°: long dashed line). For example, the average data point for the targets *x* = 10° and *y* = ± 20° (direction ± 63°), represented as a black dotted cross at eccentricity 22° (X axis), is much closer to the red dotted line associated with target direction ± 63° than to the red continuous line associated with target direction 0°. The fact that a complex logarithmic function (F_08_) takes into account both eccentricity and direction with a single parameter and provides the best fit for the data supports the hypothesis that errors made by the patients in their contralesional hemifield result from an insufficient “magnification” of a 2-dimensional surface due to the PPC lesion.

The models applied to the whole dataset were the sum of two components. The first component was the best model for controls and for the non-ataxic hemifield of patients. The second component modeled the difference between pointing errors in the non-ataxic and ataxic hemifields. The two components were fitted fully independently. For the second component, we assessed hundreds of models obtained by systematically combining four independent options. The first option was the choice of a non-linear function. Since the data in **Figure 3** suggested that the distance from the fixation point (or a close position) to the target was a non-linear function of target eccentricity that did not vary across patients, we assessed 21 different one- and two-parameters functions d = f(tgte) where d is the distance from the fixation point to the endpoint and tgte the target eccentricity (functions are listed in **Table 4** and some represented in **Figure 4**). As indicated in the legend of **Table 4**, several functions were inspired from the literature on central magnification in cortical area V1 and superior colliculus. The second option concerned different choices for the eccentricity tgte: this could correspond to the eccentricity of the target either with respect to the fixation point as in Equation 2 or with respect to a point close to it as in Equation 1. In addition to (0,0) and (X0, Y0), we assessed the points (X0 + HFB, Y0) and (X0 - HFB, Y0) as possible origins. The former was based on the hypothesis that the horizontal bias x0 and HFB in the non-ataxic hemifield also holds in the ataxic hemifield. The latter relied on the hypothesis that, as in controls, the hemifield bias (HFB) had opposite values in opposite hemifields. It should be noted that non-null horizontal biases may cause the directions of the final positions (with respect to the fixation point) to differ slightly from that of the targets. The third option concerned additive biases similar to those in the first right-hand term of Equations 1 and 2. As for the second option, but independently from it, we considered the following possibilities: (0,0), (X0,Y0), (X0 - HFB, Y0) and (X0 + HFB, Y0). The fourth option focused on the CE. On the one hand, it could be considered as a visuomotor error that had no reason to be included in the ataxic field component of the model. Alternatively, it could be considered as the result of an eccentricity-dependent incomplete transformation of a default movement toward the fixation point into a movement toward the target or its biased representation. According to this hypothesis, we also assessed whether multiplying the vector resulting from the first option by the same (1 + CE) factor as in the ipsilesional hemifield significantly improved the fitting.

**Table 4:** Non-linear functions used to model patients’ contralesional hemifield errors.

One-parameter functions		
Fo_1_	Linear	*d* = tgte × (1-E1)
F_02_	Quadratic	*d* = tgte × (1-E2 × tgte)
F_03_	Cubic	*d* = tgte × (1-E3 × tgte^2^)
F_04_	Power	*d* = tgte^POW^
F_05_	Logarithmic 1	*d* = K × log(tgte)
F_06_	Logarithmic 2	*d* = BPAR × log(l + tgte/BPAR)
F_07_	Logarithmic 3	*d* = K/150 × log(l + tgte/150)
F_08_	Logarithmic 4	d = BPAR × modulus[log(l + (tgtx + i × tgty)/BPAR)]
Two-parameters functions		
F_11_	Linear + Quadratic	*d* = tgte × (1-E1-E2 × tgte)
F_l2_	Linear + Cubic	*d* = tgte × (1-El-E3 × tgte^2^)
F_l3_	Quadratic + Cubic	*d* = tgte × (1-E2 × tgte-E3 × tgte^2^)
F_l4_	Power (1 + tgte/b)	*d* = BPAR^(1-^^POW)^ × [(1 + tgte/BPAR)^POW^ -1]
F_l5_	Logarithmic 5	*d* = K × log(l + tgte/BPAR)
F_l6_	Logarithmic 6	*d* = K × modulus[log(l + (tgtx + i × tgty) / BPAR)]

F_21_ to F_27_: As F_01_ to F_08_ except F_07_, with individual variations of the single parameter around a population value		


*The table lists the linear and non-linear models used to fit d (the distance from the fixation point to the endpoint) as a function of target eccentricity tgte in the ataxic field. Parameters are indicated in uppercase. The logarithmic functions F_06_–F_08_ are inspired from the literature on cortical magnification (e.g., Polimeni et al., 2006; Schira et al., 2007) where BPAR is a parameter characterizing the shape of the peripheral part of the dipole model of area V1 (Balasubramanian et al., 2002). In the function F_07_ the parameter BPAR is replaced by 150°, as in Schira et al. (2007), so as to introduce the scale parameter K which is also present in the dipole model. We disregarded the third parameter of the dipole model because it characterizes the shape of the foveal part and has little importance for peripheral vision. The function F_08_ is based on the complex logarithm formulation of the dipole model where tgtx and tgty are the horizontal and vertical coordinates of the point of eccentricity tgte. This function yields values similar to F_06_ except that it slightly increases *d* as target direction moves away from the horizontal axis along iso-eccentric curves. The power function F_14_ yield results similar to F_06_ as p tends toward 0. The test of F_14_ in addition to F_06_ was motivated by the fact that some studies suggested that the magnification factor could be equal to the integral of (tgte + BPAR)-(1-POW) (e.g., Duncan and Boynton, 2003) while dipole models were based on a complex logarithm, i.e., on the integral of a magnification factor equal to (tgte + BPAR)-1. F_15_ and F_16_ are versions of F_06_ and F_08_ allowing for dissociating the scale parameter K from the shape parameter BPAR as in models of cortical areas. In functions F_21_ to F_27_ the single constant parameter of functions F_01_–F_06_ and F_08_ is complemented with a parameter defining its standard deviation across patients.*

The independent assessments of all four options yielded clear results. As the three last options provided similar results with most eccentricity functions, we first fitted the former and then the quantitative functions of eccentricity. First (fourth option), introducing the contraction factor in the ataxic component of the model strongly increased the log-likelihood (LLR) by 22 (median across eccentricity functions), reduced the BIC by 45 and decreased the sum of square residual errors (SSR) by 6%. This fitting improvement could not be tested because it did not require any additional parameters. However, its unlikelihood can be better appreciated considering that in the present modeling an additional parameter that reduces the LLR by 8 or 10 was significant with probability equal to 10^-4^ and 10^-7^, respectively. Similarly, it is unlikely that a single random parameter reduces a sum of 336 square residuals errors by more than 1%. Therefore, we can conclude that the same contraction bias characterizes the errors in both the healthy and ataxic hemifields. As the contraction bias varies across individuals (and across hands), in contrast with the parameters of most eccentricity functions, this result shows that those individuals who display larger biases toward the fixation point in the ipsilesional hemifield will also do so in the opposite hemifield (e.g., ‘kod,’ **Figure 1**).

The results were still more clear-cut for the additive biases (third option). With respect to an absence of bias, introducing in the ataxic hemifield component the same additive (X0,Y0) bias as in the healthy hemifield increased the LLR by 60, decreased the BIC by 120, and reduced the SSR by more than 20%. These figures demonstrate beyond any doubt that the same systematic biases that affected the pointing in the healthy hemifield also caused large errors in the ataxic hemifield. Patients that tended to make leftward or upward errors (e.g., ‘suz’) in their healthy hemifield tended also to do so in their ataxic hemifield. The results for the hemifield bias contrasted with those of the horizontal and vertical biases. Indeed, adding or subtracting the constant HFB value did not yield consistent increases of LLR (medians across eccentricity functions for – HFB and + HFB: +4.8 and +3.0). This leads to the conclusion that the hemifield bias HFB, in sharp contrast with its high significance in controls and the healthy hemifield of patients, explains little if any of the errors in the ataxic hemifield.

The analyses also provided clear-cut results concerning the introduction of the horizontal, vertical, and hemifield biases into the functions fitting the eccentricity of final positions (second option). Using the eccentricity of targets with respect to a biased origin at (X0, Y0) in the eccentricity functions improved the LLR (-15.6), the BIC (+31) and the SSR (+4.1%). By contrast, using biased origins at (X0-HFB, Y0) or (X0+HFB, Y0) worsened the likelihood (+6 and +10.5). These results suggest again that the hemifield bias is a specific characteristic of pointing in the healthy hemifield, while the horizontal and vertical biases influence the pointing similarly in both hemifields.

In order to compare the eccentricity functions in an unbiased way, we first focused on improving the variance function of the mixed-effect models (In ME models, the variance function makes the residual errors independent of factors such as the hemifield or the target distance). We found that the variance of the residual errors tended to increase faster with target eccentricity in the ataxic than in the healthy hemifield (LLR ratio varying from 33 to 96 across functions, all *p*-values equal to 0). According to the fitted coefficient of the exponential, the logarithm of the standard deviation of residual error linearly increased with target eccentricity with a slope varying between 0.028 and 0.030. After taking into account this difference, the statistical tests did not reveal any significant hemifield-dependent difference in variance size. Similarly, the slopes did not display any significant difference for the ataxic or healthy hands in the ataxic hemifield or in the healthy hemifield.

**FIGURE 5:**
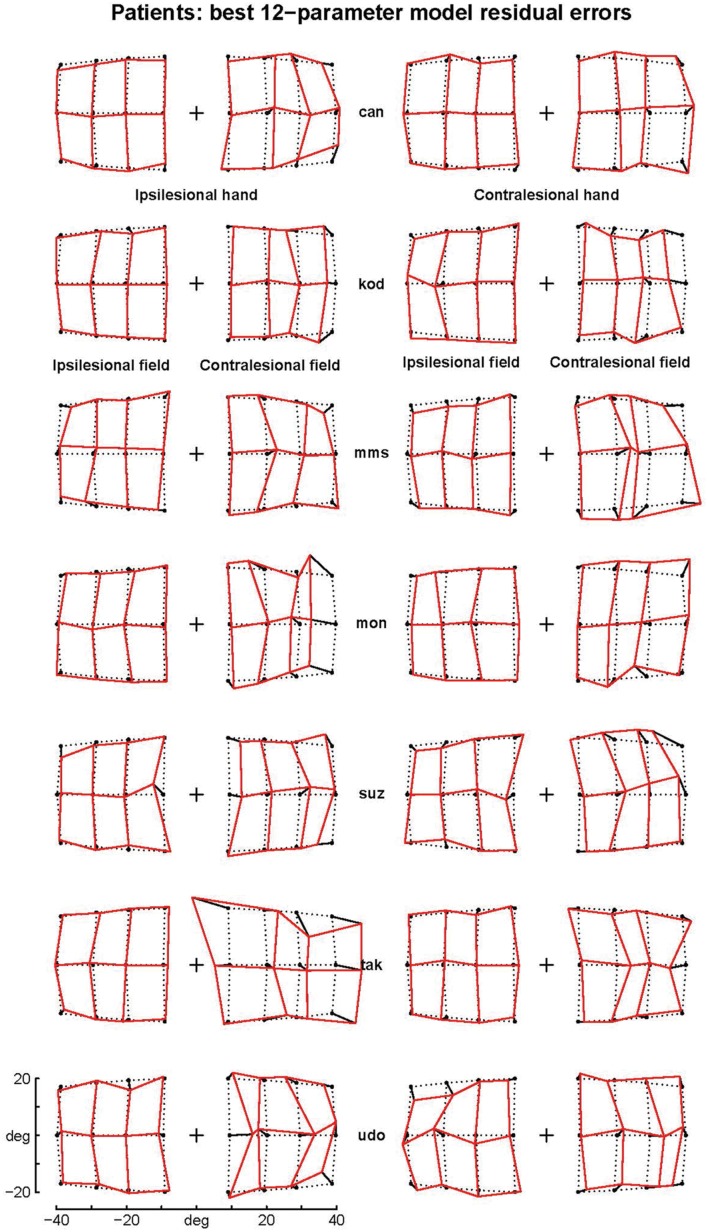
**Residual errors of the complex logarithmic model F_08_.** F_08_ includes the best one-parameter model of the errors specifically associated with pointings in the ataxic hemifield [equation d = BPAR × modulus(log(1 + (tgtx+i × tgty)/BPAR)]. Residual errors are represented as segments starting from target positions (black points). Comparison with **Figure 1** shows that residual errors are small with respect to patients’ raw errors in the contralesional hemifield. The residual errors are also strongly reduced in the ipsilesional hemifield because the F_08_ model also includes components fitting errors in the ipsilesional hemifield and errors associated with the contralesional hand.

**FIGURE 6:**
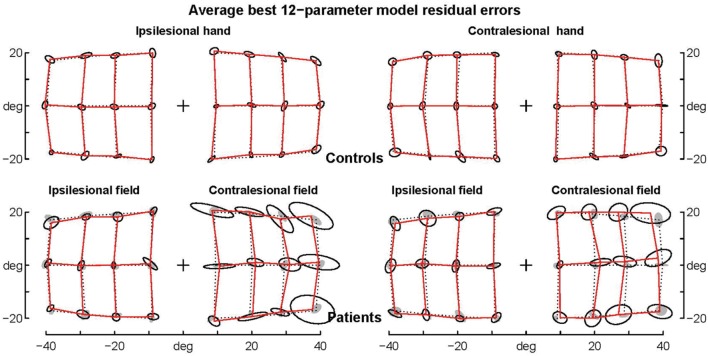
**Average residual errors of the model F_08_ for controls and patients.** Average residual errors are shown for controls **(top)** and patients **(bottom)** together with the ellipses of inter-individual variability. Controls’ ellipses are also shown as shadows in the patients’ error grid. Note that with few exceptions the average residual errors of patients are within the controls’ ellipses.

All eccentricity functions reduced the errors in a highly significant way (first option). With one parameter, the best-fitting function was F_08_ (see **Figure 4**), based on a complex logarithm. It reduced the sum of square errors in the ataxic field by 83.1% (from 13126 to 2224 deg^2^). Its residual errors are shown in **Figure 5** (individuals) and 6 (average). The average residual errors of function F_08_ (**Figure 6**) are considerably smaller than the raw errors (**Figure 2**). The next best functions were F_06_ and F_07_, also based on logarithms, followed by the cubic F_03_. With respect to F_08_, they display LLR decreases of 6.2, 19.7, and 26.1, respectively (+1.0 to +1.3% of SSR). Concerning the best two-parameter functions, the best ones were the power-based F_14_, the logarithm-based F_15_, and the combination of linear and quadratic errors F_11_. Although they reduced the SSR only slightly more than the one-parameter functions (83.5–83.7%), they performed significantly better than the best one-parameter function according to a LLR ratio test (Comparison between F_08_ and F_14_: LLR = 17.8, *p* = 2.4 × 10^-5^). The fitted parameters of some of the best functions are displayed in **Table 5.** It can be seen that they have relatively narrow confidence intervals, which shows a good fitting of the data. It should also be noted that the value of the p parameter of the power-based function F_14_ (-0.07) is close to zero, which means that the function is close to the logarithmic-based F_06_ (and F_08_) functions. More precisely, the latter is the integral of (1+E/70.1)-1 and the former the integral of (1+E/28.0)-1.076 where E is the target eccentricity. In conclusion, as shown in **Figure 4**, the complex logarithmic function F_08_ provides the best fitting because it takes into account both eccentricity and direction (with a single parameter).

**Table 5:** Parameter values for the best fitting models of errors in the ataxic hemifield.

	F_08_ (complex log)		F_06_ (log)	F_14_ (power close to log)	
			
	Value	Confid. Interv	Value	Value	Confid. Interv
**Constant (fixed) parameters**					
Contraction error (fix ed)	-0.037	[-0.044, -0.029]	-0.037	-0.039	[-0.047, -0.031]
Hemifield bias HF B (fix ed)	0.845	[0.709, 0.981]	0.839	0.817	[0.709, 0.981]
Shape parameter BPAR	60.80	[53.1, 68.5]	70.09	28.02	[53.1, 68.5]
Exponent POW				-0.076	
**Standard deviation parameters**					
Contraction error (non-ataxic hand)	0.010	[0.006, 0.017]	0.010	0.011	[0.006, 0.017]
Contraction error (ataxic hand)	0.058	[0.033, 0.101]	0.058	0.061	[0.033, 0.101]
Horiz. bias (right hand)	0.612	[0.394, 0.949]	0.608	0.701	[0.394, 0.949]
Horiz. bias (left hand)	0.444	[0.266, 0742]	0.435	0.352	[0.266, 0742]
Vertical bias	0.656	[0.441, 0.949]	0.660	0.660	[0.441, 0.949]
Ex pon. variance ratio	1.475	[1.341, 1.622]	1.475	1.464	[1.341, 1.622]
Ex pon. variance slope (healthy)	0.007	[0.003, 0.012]	0.007	0.008	[0.003, 0.012]
Ex pon. variance slope (ataxic hemifield)	0.029	[0.024, 0.034]	0.030	0.029	[0.024, 0.034]


*Fitted values for the parameters of the models F_08_, F_06_, and F_14_ (see Equations in **Table 4**).*

Finally, we made sure that the above results did not depend on outlying errors. More precisely, as the errors of patient ‘tak’ when pointing with the healthy hand in the ataxic hemifield displayed a pattern that was very different from the other patients (see **Figure 1**), we removed this outlier set of 12 errors and refitted the 21 functions with the three same options (Mixed-effects modeling allows such a procedure). We again found the same best one-parameter functions (F_08_ followed by F_06_, F_03_, and F_07_, with 8.6, 24.8 and 28.1 LLR decrease) and the same best two-parameter functions (F_14_ followed by F_15_ and F_11_ with -0.1 and 1.5 of LLR difference). The best two-parameter function remained significantly better than the best one-parameter function (LLR = 29.7, *p* = 5.1 × 10^-8^). In addition, the large reduction of the sum of residual errors for pointings with the healthy hand in the ataxic hemifield (45%) confirmed that the patient ‘tak’ may not point like other patients for this hand-hemifield combination.

### Comparison between Central Magnification Distorsions and Ataxic Hemifield-Specific Errors

Complex logarithmic functions provide the best fitting of the distortions of the whole space representation which accompanies central magnification observed in monkey and human visual maps. Models derived from small eccentricities (Polimeni et al., 2006; Schira et al., 2007) predict an inversion of the curvature of isoeccentricity rings (Figure 8 in Polimeni et al., 2006; Figure 6D in Schira et al., 2007). However, this inversion does not correspond to the observed topography of visual area V1 (Horton and Hoyt, 1991) and is not predicted by other models based on eccentric points up to 60° (Figure 3 in Ottes et al., 1986 for the superior colliculus; Figure 9 in Wu et al., 2012 for the occipital cortex).

**FIGURE 7:**
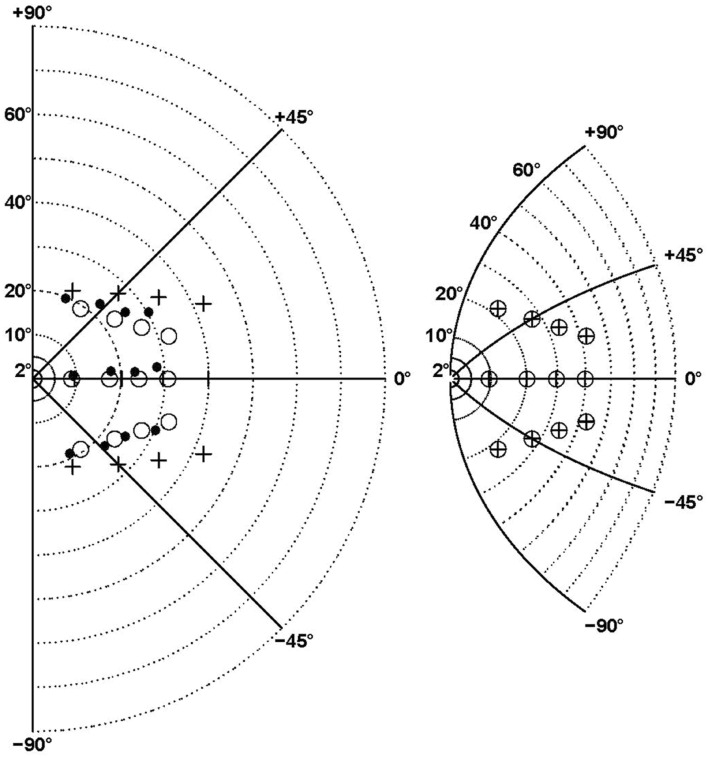
**Comparison between central magnification distorsion associated with the best model (F_08_) and ataxic hemifield-specific errors.** The right panel shows the transformation of the right hemifield (left panel) by the complex logarithmic function z’ = b^∗^log(1+z/b) where z’ and z are complex representations of points in 2D space and b is equal to the F_08_ model fitted value (BPAR = 60°8). The isopolar rays isoeccentricity rings are shown by continuous and dotted lines, respectively. The targets are indicated by crosses **(left panel)** and crossed circles **(right panel)**. In the **(left panel)** the black points represent the final positions averaged across patients for the contralesional hemifield, while the open circles are obtained by translating the pattern of transformed targets shown in the **(right panel)**. Note that here the transformation is based only on the b parameter. The circles and points would almost coincide as in **Figure 6** if all the F_08_ model fitted parameters were taken into account.

In order to facilitate the comparison between our results and models of central magnification, we transformed the visual hemifield with a complex logarithmic function using the model-fitted value BPAR from the F_08_ function that described the additional errors in the ataxic hemifield (**Figure 7**). The targets transformed in this way (crossed circles in right panel) were then superimposed on the left panel of **Figure 7**, in order to be compared with the final positions reached by the patients (black dots, left panel). This shows that patients’ final positions are similar to the transformed targets and therefore reflect the metric of the complex logarithmic representation. Furthermore, the transformed targets would almost exactly coincide with the final positions (black points, left panel) if the other parameters of the F_08_ model were taken into account. This is clear from **Figure 6** which shows that patients’ final positions can be almost perfectly transformed into target positions, likewise the inverse transformation could almost perfectly turn the pattern of targets into the pattern of patients’ final positions.

## Discussion

Here, we conducted data-driven modeling of an exceptionally large sample of unilateral optic ataxia patients (seven patients) and seven control subjects pointing with both hands to 24 peri- pheral targets (12 in each visual hemifield) while fixating a central position (labeled F on **Figure 4**, Blangero et al., 2010). The aim of analysis was to deconstruct the patients’ spatial errors, i.e., to model the source of their errors with as few parameters as possible.

The most important result of this study is that the large pointing errors made by patients with optic ataxia in their contralesional hemifield are best fitted by one- or two-parameter complex logarithmic functions. Complex logarithmic functions have often been used for modeling the transformation from visual hemifield onto cortical and collicular surfaces (Schwartz, 1980; Ottes et al., 1986; Van Gisbergen et al., 1987; Polimeni et al., 2006; Schira et al., 2007; Wu et al., 2012). The best fitting of these optic ataxia field effect errors was provided by the complex logarithmic function F_08_ because it takes into account the two dimensions of target location in space (both eccentricity and direction, see **Figure 4**). This supports the hypothesis developed in two steps below that when the PPC is unilaterally damaged, pointing errors made by the patients in their contralesional hemifield (whatever the hand) result from the use of a vicarious visuomotor interface characterized by the footprint of central magnification (a 2-dimensional map with insufficient “magnification” of contralateral peripheral visual space).

First, it suggests that information about target positions in the contralesional hemifield comes exclusively from areas with a magnification of central vision. Possible areas include V1, or other occipito-temporal areas of the ventral visual stream which have been shown to be potentially involved in visual reaching (Jeannerod et al., 1994; Milner et al., 1999; review in Pisella et al., 2006; more direct evidence from Hesse and Schenk, 2014), and the superior colliculus which has been shown to be involved in limb reaching movements in the cat (Courjon et al., 2004) and in monkeys (Stuphorn et al., 2000). Indeed, both occipito-temporal and collicular maps have a logarithmic representation of visual space and are connected to frontal and parietal areas involved in visuomotor control.

Second, this finding suggests that pointing errors in optic ataxia arise from the destruction of an area without the spatial under-representation of peripheral vision that accompanies central magnification and/or of an area implementing the crucial transformations necessary to compensate for central magnification distortions. Several reasons indicate that a likely candidate is the human homologue of monkey area V6A. First, like macaque V6A, human V6A has an over-representation of the periphery (Galletti et al., 1996, 1999; Pitzalis et al., 2013) which could mean that this area has little or no central magnification. Furthermore, to our knowledge, this is the only visual area displaying this property. Second, this area is lesioned in patients with optic ataxia. Indeed, when optic ataxia is defined as misreaching toward the contralesional hemifield, the lesion overlap involves the parieto-occipital junction in its lateral and medial parts (Karnath and Perenin, 2005; Pisella et al., 2009; Blangero et al., 2010), close to the location of the human homologue of V6A (Pitzalis et al., 2013). Third, functional magnetic resonance imaging and transcranial magnetic stimulation studies have shown that this region is involved in pointing toward peripheral targets (Prado et al., 2005; Vesia et al., 2010; Pitzalis et al., 2013; Rossit et al., 2013).

One possible interpretation of this result is that hand movements toward peripheral targets are planned by modifying a default movement aimed toward the fixation point (as proposed by Pisella et al., 2009). Indeed, several studies have already concluded that movements are planned from a default movement (Hening et al., 1988; Ghez et al., 1997; Pellizzer and Hedges, 2004; Prado et al., 2005). Moreover, Chang and Snyder stated that in the parietal reach region “eye and hand gain fields are systematically arranged within each individual neuron to form a compound gain field that encodes the distance between the point of the fixation and hand position” (Chang et al., 2009; Chang and Snyder, 2010). Accordingly, magnetic misreaching (Carey et al., 1997) has been described as a condition where the patient cannot point elsewhere than where he is fixating. In some optic ataxia patients we have observed this magnetic misreaching in the acute phase. Thus, magnetic misreaching may simply result from a lesion of the human V6A which has been demonstrated to transform the hand-gaze vector (default movement toward the fixation point elaborated in the parietal reach region) into a hand-target vector (Hadjidimitrakis et al., 2014; Bosco et al., 2015, 2016). In the absence of human V6A, in the chronic phase, the system may use vicarious cortical or collicular maps characterized by the footprint of central magnification. In these maps, the same constant cortical (or collicular) distance may correspond to 5° of external space close to foveal representation, to 10° further away and to 20° in far periphery. The observed metrics of saccades elicited by electrical stimulation of the colliculus in monkeys have been shown to reflect this logarithmic distortion of target eccentricity (Ottes et al., 1986). Here, the idea is that a given distance away from the foveal representation in the cortical map would yield a proportional modification of the default movement toward the peripheral target. In control subjects, the small but highly significant hypometria with respect to the fixation point suggests that the modification of the default movement is not fully implemented. In optic ataxia patients, this small hypometria is still present, but in addition, the modification of the default movement appears to rely on a cortical map with central magnification.

### Summary

These findings are important because they are the first to suggest that the PPC has a specific role in correcting for the structural organization of the retina and its consequence on the neural representation of visual space. Our hypothesis is that only that part of the PPC associated with optic ataxia field effect errors has a neural representation of visual space without the distortions that accompany central magnification. It remains to be tested whether this interface is used for visuo-motor integration only, or whether it is also used for fine visual discrimination on a large spatial scale, as suggested by the deficit of optic ataxia patients in visuo-spatial perception (Pisella et al., 2013). This would be consistent with neuroimaging data showing the involvement of SPOC in coding intrinsic (size) and extrinsic (location) object properties not only in grasping, but also in passive viewing conditions (Faillenot et al., 1997; Monaco et al., 2015).

Our analyses and modeling confirm the view that one main difficulty in planning and controlling hand movements is the non-linear transformations associated with the 3-D geometry of the eye-head-reach systems (Crawford et al., 2011). These findings also complement previous views of optic ataxia. They support the hypothesis that optic ataxia is the result of the lesion of a ‘human’ area V6A (Galletti et al., 2003). They are also in line with the view that within the PPC there are anterior and lateral areas computing hand location in eye-centered coordinates and posterior and medial regions computing target location in eye-centered coordinates (Pisella et al., 2009) and that these two types of information can be directly combined (Buneo et al., 2002; Buneo and Andersen, 2006; Hadjidimitrakis et al., 2014; Bosco et al., 2015, 2016).

## Author Contributions

PV analyzed the data. HO collected the data. AB made a first analysis of the data for the 2010 paper and began to think at their interpretation. PV, KR, and LP wrote the paper. AB and YR contributed to theoretical discussions.

## Conflict of Interest Statement

The authors declare that the research was conducted in the absence of any commercial or financial relationships that could be construed as a potential conflict of interest.
